# Diseases of the Lungs and Pleuræ

**Published:** 1896-01-04

**Authors:** 


### MEDICAL PROGRESS AND HOSPITAL CLINICS.

[*The Editor will be glad to receive offers of co-operation and contributions from members of the profession. All letter should be addressed to* THE EDITOR, AT THE OFFICE, 428, STRAND, LONDON, W.C.]

### PROGRESS IN MEDICINE.

### DISEASES OF THE LUNGS AND PLEURÆ.

Paul Paquin's investigations on the effect of horse-serum in tuberculosis are also referred to by Wordin. He uses the blood-serum of the horse because this animal is immune to the bacillus tuberculosis. Ten drops are hypodermically injected between the shoulder-blades, and in a few days this is increased to twenty drops, then thirty, forty, sixty. No reaction follows, and there is no pain. He summarises his conclusions, after some years of investigation, as follows: "(*a*) Serum-therapy in tuberculosis is efficacious; (*b*) horse blood-serum may be rendered strongly antagonistic to the germ by the proper treatment of the animals; (*c*) a horse properly treated three months may yield serum with immunising power that will probably prove sufficient to arrest consumption in the first stages in three or four months, and sometimes in less time, and in the second stage in four to six months." Hewetson[7] reports negative results of Paquin's serum in experimental tuberculosis. Obtaining a supply through Messrs. Heath and Beatty, he inoculated seven guinea-pigs in the right groin with ·75 c.c. of solution of tuberculous spleen. Five test guinea-pigs received daily 6 minims of Paquin's solution to time of death, except for six days, when they were given on alternate days. The five test animals lived on an average for 50·8 days; the two control animals for 58·5 days. He found that the lesions in no way differed from those usually observed in tubercular guinea-pigs, nor were there any differences to be noted between the lesions of the control and test animals. The results certainly seem to justify one in concluding that anti-tubercle serum has no influence in prolonging the life of tubercular guinea-pigs.

The results of the necropsy in a case treated by Koch's tuberculin are record by Sinclair Coghill. He first calls to mind his detailed report, in 1891, of the successful treatment of ten cases of pronounced pulmonary tuberculosis by Koch's method. The subsequent history of these cases has been carefully noted, and reports of their condition obtained at frequent intervals from competent observers. At present, after a lapse of over four years, eight of these ten patients are in excellent health, with every evidence of complete arrest of the pulmonary lesion, and are following their ordinary avocations. One died eighteen months after leaving hospital from symptoms due to fæcal accumulation. The remaining case is the subject of his present report.

The patient was inoculated, almost under protest, both lungs being affected, the tuberculous process acute, advanced, and extensive, and the general conditions also most unfavourable. The case was kept under observation till his death from cardiac failure on November 17th, 1894. With many of the usual concomitant symptoms and signs of rapidly-advancing phthisis, he presented at the outset the following signs: On the right side in front the percussion note was impaired to the third rib, with expiration prolonged, vocal resonance increased, and distant amphoric breathing under the clavicle. Behind, the note was impaired at the apex, expiration was lengthened, and increased vocal resonance to spine of scapula. In front of the left lung there was absolute dulness to the third rib and impaired to the fifth rib. There was loud superficial, cavernous breathing, with pectoriloquy to the lower margin of the third rib, with moist crepitations over the whole lung. Posteriorly there was absolute dulness at the apex, and in the supraspinous fossa; in the latter there was amphoric breathing, but no crepitation. At the post-mortem the body was fairly well nourished. The whole of the upper lobe of the left lung was occupied by a cavity with thick fibrous capsule, and lined by a pyogenic membrane with pus-moistened walls. The middle lobe was adherent to the upper, and partly excavated, the lining capsule presenting characters similar to the upper cavity. The right lung contained a caseous nodule, surrounded by a thick fibrous wall. The whole upper third of this lung was occupied by a cavity with an extremely thick fibrous wall. He remarks that if the physical signs and symptoms of this patient, as ascertained by competent observers before treatment, the phenomena developed during its course, and his condition at its termination, and subsequently, be compared with the state of the lungs as revealed at the necropsy, there can hardly be any hesitation in ascribing the arrest of this extensive area of acute pulmonary tuberculosis to judicious tuberculin inoculation. This case confirms him in the opinion he has always held, and still holds, that tuberculin has potent therapeutic efficacy in the treatment of tuberculosis when used judiciously and with caution in doses and under conditions adapted to each individual case. On the Continent, Koch's method has survived the haste which too prematurely condemned it. Finally, he states that no less an authority than Armand Ruffer has informed him that he was much impressed with what he saw of this treatment on the occasion of a recent visit to Berlin.

McGillicuddy[8] insists on the value of diet and systematic muscular exercise in the treatment of tuberculosis, and argues that poor or sterilised granite or trap-rock waters, taken hot in sufficient quantities and at proper times, combined with systematic muscular exercise, hot bath and massage, will certainly help to cleanse out the tissues of the body. We may furthermore take advantage of the bactericidal properties of the blood-serum. The plan of treatment the author has instituted consists in giving at rather frequent intervals a considerable quantity of carefully roasted or boiled beef or mutton, raw eggs, stale bread, butter, sterilised milk, and vegetables. After a few days of treatment the meat should not be less in amount than a pound a day, and the quantity of bread and vegetables should be even, if possible, somewhat larger. He then proceeds to describe and detail the kinds of exercise he would advocate for developing the muscles, broadening the chest, and deepening inspiration.

[7] N. Y. Med. Journ., Nov. 9, 1895. [8] Med. Record, Nov. 9,1895.

